# Tissue Proteome of 2-Hydroxyacyl-CoA Lyase Deficient Mice Reveals Peroxisome Proliferation and Activation of ω-Oxidation

**DOI:** 10.3390/ijms23020987

**Published:** 2022-01-17

**Authors:** Youssef Khalil, Sara Carrino, Fujun Lin, Anna Ferlin, Heena V. Lad, Francesca Mazzacuva, Sara Falcone, Natalie Rivers, Gareth Banks, Danilo Concas, Carlos Aguilar, Andrew R. Haynes, Andy Blease, Thomas Nicol, Raya Al-Shawi, Wendy Heywood, Paul Potter, Kevin Mills, Daniel P. Gale, Peter T. Clayton

**Affiliations:** 1 Genetics and Genomic Medicine, Great Ormond Street Institute of Child Health, University College London, London WC1N 1EH, UK; y.khalil@ucl.ac.uk (Y.K.); sara.carrino@studio.unibo.it (S.C.); f.mazzacuva@uel.ac.uk (F.M.); wendy.heywood@ucl.ac.uk (W.H.); kevin.mills@ucl.ac.uk (K.M.); 2 Department of Experimental, Diagnostic and Specialty Medicine, University of Bologna, 40138 Bologna, Italy; 3 Department of Renal Medicine, University College London, London NW3 2PF, UK; linfujun@xinhuamed.com.cn (F.L.); a.ferlin@rbht.nhs.uk (A.F.); d.gale@ucl.ac.uk (D.P.G.); 4 Department of Nephrology, Xin Hua Hospital, School of Medicine, Shanghai Jiao Tong University, Shanghai 200082, China; 5 Clinical Genetics and Genomics Laboratory, Royal Brompton Hospital, London SW3 6NP, UK; 6 MRC Harwell Institute, Harwell Campus, Oxfordshire OX11 0RD, UK; h.lad@har.mrc.ac.uk (H.V.L.); sfalcone@well.ox.ac.uk (S.F.); n.rivers@har.mrc.ac.uk (N.R.); g.banks@har.mrc.ac.uk (G.B.); d.concas@har.mrc.ac.uk (D.C.); c.aguilar@har.mrc.ac.uk (C.A.); a.haynes@har.mrc.ac.uk (A.R.H.); a.blease@har.mrc.ac.uk (A.B.); tnicol@well.ox.ac.uk (T.N.); ppotter@brookes.ac.uk (P.P.); 7 Department of Bioscience, University of East London, London E15 4LZ, UK; 8 Genetics Unit and Wolfson Drug Discovery Unit, Centre for Amyloidosis and Acute Phase Proteins, University College London, London NW3 2PF, UK; r.al-shawi@ucl.ac.uk

**Keywords:** peroxisomes, PPARs, liver, proteomics, isoprenoids

## Abstract

Peroxisomal fatty acid α-oxidation is an essential pathway for the degradation of β-carbon methylated fatty acids such as phytanic acid. One enzyme in this pathway is 2-hydroxyacyl CoA lyase (HACL1), which is responsible for the cleavage of 2-hydroxyphytanoyl-CoA into pristanal and formyl-CoA. Hacl1 deficient mice do not present with a severe phenotype, unlike mice deficient in other α-oxidation enzymes such as phytanoyl-CoA hydroxylase deficiency (Refsum disease) in which neuropathy and ataxia are present. Tissues from wild-type and *Hacl1*^−/−^ mice fed a high phytol diet were obtained for proteomic and lipidomic analysis. There was no phenotype observed in these mice. Liver, brain, and kidney tissues underwent trypsin digestion for untargeted proteomic liquid chromatography-mass spectrometry analysis, while liver tissues also underwent fatty acid hydrolysis, extraction, and derivatisation for fatty acid gas chromatography-mass spectrometry analysis. The liver fatty acid profile demonstrated an accumulation of phytanic and 2-hydroxyphytanic acid in the *Hacl1*^−/−^ liver and significant decrease in heptadecanoic acid. The liver proteome showed a significant decrease in the abundance of Hacl1 and a significant increase in the abundance of proteins involved in PPAR signalling, peroxisome proliferation, and omega oxidation, particularly Cyp4a10 and Cyp4a14. In addition, the pathway associated with arachidonic acid metabolism was affected; Cyp2c55 was upregulated and Cyp4f14 and Cyp2b9 were downregulated. The kidney proteome revealed fewer significantly upregulated peroxisomal proteins and the brain proteome was not significantly different in *Hacl1*^−/−^ mice. This study demonstrates the powerful insight brought by proteomic and metabolomic profiling of *Hacl1*^−/−^ mice in better understanding disease mechanism in fatty acid α-oxidation disorders.

## 1. Introduction

**Figure 1:**
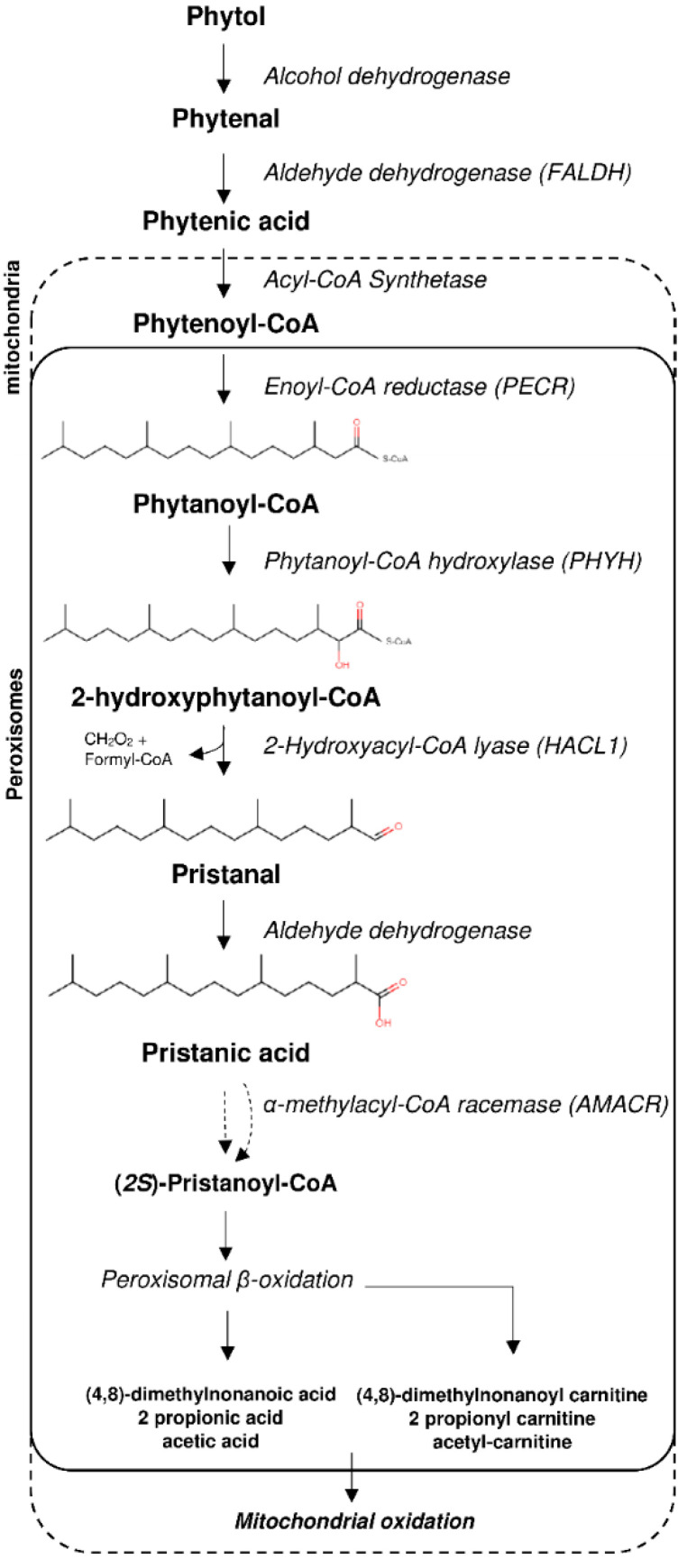
Phytol degradation pathway via peroxisomal alpha-oxidation.

The primary mechanism for fatty acid degradation involves β-oxidation, in which a multistep enzymatic pathway converts fatty acid chains into acetyl-CoAs and 2-carbon shorter fatty acyl-CoAs which can in turn undergo further β-oxidation. This mechanism is prevented in the presence of a methyl group on the β-carbon such as with the 3-methyl-branched phytanic acid (3,7,11,15-tetramethyl hexadecanoic acid). In such case, a preceding peroxisomal α-oxidation pathway is required in order to shorten phytanic acid by one carbon into pristanic acid (2,6,10,14-tetramethylpentadecanoic acid) which can then undergo β-oxidation (Figure 1). The fatty acid α-oxidation pathway involves activation of phytanic acid to phytanoyl-CoA, hydroxylation by phytanoyl-CoA 2-hydroxylase (PHYH), cleavage of 2-hydroxyphytanoyl-CoA into pristanal and formyl-CoA by 2-hydroxyacyl CoA lyase (HACL1), and oxidation of pristanal to pristanic acid by an aldehyde dehydrogenase. Furthermore, pristanic acid with a C-2 in an *(R)*-configuration requires conversation to an *(S)*-configuration by α-methylacyl-CoA racemase (AMACR) in order to then undergo β-oxidation [1]. Another pathway for fatty acid degradation is through ω-oxidation, in which dicarboxylic acids are formed and subsequently undergo β-oxidation from the omega end [2]. This pathway is catalysed by CYP450 enzymes and the peroxisomal β-oxidation pathway which are regulated by peroxisome-proliferator-activated receptor α (PPARα) [3,4].

The phenotype of HACL1 deficiency in human (if any) has yet to be delineated but other inborn errors in the α-oxidation pathway have been identified. Phytanoyl-CoA 2-hydroxylase deficiency, known as (adult) Refsum disease, leads to phytanic acid accumulation and affected individuals develop neuropathy, ataxia, and retinitis pigmentosa [5]. Since phytanic acid is strictly exogenous, mainly derived from ruminant animals and fish [6], patients who undergo dietary restriction of phytanic acid can lower their plasma phytanic acid levels, and reduce some of the clinical manifestations [7,8]. A similar phenotype is manifested in patients with AMACR deficiency, in which pristanic acid and phytanic acid accumulate [5]. The mouse model of Refsum disease on a high phytol diet presents with a phenotype similar to that of humans, including neuropathy and ataxia, along with accumulation of phytanic acid [9]. Amacr-deficient mice fed a high phytol diet show an accumulation of pristanic and phytanic acid and a significantly reduced lifespan as a result of hepatic failure, renal dysfunction, and brain lesions [10]. In contrast, *Hacl1* knockout mice do not develop neurological disease when fed a high phytol diet; rather, in one study, they show weight loss, absence of abdominal white adipose tissue, an enlarged mottled liver, and reduced hepatic glycogen and triglycerides [11]. Immunohistostaining of mouse *Hacl1*^−/−^ liver reveals the induction of the PPARα-target CYP4A1 involved in the ω-oxidation pathway [11]. This alternative pathway and evidence of a yet unidentified endoplasmic reticulum lyase in the central nervous system [11] may explain the lack of a severe phenotype.

To date, there has only been one study published that shows evidence of PPARα activation in *Hacl1*^−/−^ mouse liver [11]. Here, we performed proteomic analyses on *Hacl1*^−/−^ mouse liver, kidney and brain after feeding on a high phytol diet. We identified tens of proteins that were up- or downregulated in the liver, providing more insight on the activation of the ω-oxidation alternative pathway and other pathways regulated by PPARα.

## 2. Materials and Methods

### 2.1. Animals

A transgenic mouse strain (C57BL/6NTac-Hacl1tm1a(KOMP)Wtsi) harbouring the ‘knockout-first’ conditional cassette [12] in the *Hacl1* gene was obtained from the European Mutant Mouse Archive. At week 3 after birth, 4 groups (male knockout (*Hacl1*^−/−^), male wild-type (*Hacl1^+^*^/*+*^), female knockout (*Hacl1*^−/−^) and female wild-type (*Hacl1^+^*^/*+*^) each comprising 10 individuals were weaned onto standard compressed Western diet that was replaced with phytol-enriched (0.2% *v*/*v*) Western diet (both obtained from Research Diets Inc., New Brunswick, NJ, USA,) at week 6. Over a further 6-week period, body weight, markers of renal function, acoustic brainstem, fear conditioning, locomotor activity responses and optical coherence tomography were performed as previously described [13]. Tissues were isolated immediately after death and blotted proteins were probed with rabbit polyclonal antibodies (Cat. LS-C137258) obtained from LSBio (Seattle, WA, USA). Mice were maintained in the Mary Lyon Centre in Harwell UK, in specific pathogen-free conditions, with environmental conditions as outlined in the Home Office Code of Practice. Home Office ethical approval was granted under project licence 30/3070 and mice were euthanized by Home Office Schedule 1 methods. Welfare end points included chronic weight loss exceeding 20%, or rapid weight loss of no more than 15%, and excessive urination as well as general indicators of health. All procedures were carried out according to UK Home Office regulations, those laid out in the project license (30/3070), and local ethical guidelines.

### 2.2. Materials

Phytanic acid, urea, thiourea, iodoacetamide, 1,4-Dithioerythritol, Tris base, non-ionic detergent octylphenoxy poly(ethyleneoxy)ethanol (IGEPAL^®^ CA-630), and zwitterionic detergent Amidosulfobetaine-14 (ASB-14) were purchased from Sigma-Aldrich. LysC/Trypsin was purchased from Promega (UK). MTBSTFA + 1%TBDMS (N-Methyl-N-(tert-butyldimethylsilyl)trifluoroacetamide, Tertbutyldimetheylchlorosilane) was purchased from Thermofisher Scientific. Heptadecanoic-17,17,17-d3 acid was purchased from Qmx laboratories Ltd. (Essex, UK). 2-hydroxyphytanic acid was purchased from LGC (Middlesex, UK). All proteomic solvents were of UPLC grade. All lipid analysis solvents were of HPLC grade. All other reagents were of analytical grade.

### 2.3. Preparation of Mouse Liver for Fatty Acid Analysis

Fresh frozen liver tissues (wild-type *n* = 5; *Hacl1^−^*^/*−*^ *n* = 5) were weighed and 120 mg tissue were added to 1 mL homogenisation medium containing 50 mmol/L ammonium bicarbonate + 2% IGEPAL CA-630. Tissues were then homogenised using a Minilys tissue homogeniser (Bertin Instruments, Montigny-le-Bretonneux, France) for 4x30 s intervals while placed on ice for 30 s in between intervals. The homogenates were stored at −80 °C for later analysis.

### 2.4. Fatty Acid Extraction and Derivatisation

Two hundred microliters of tissue homogenate were mixed with 5 µL of 50 µmol/L heptadecanoic-17,17,17-d3 acid as an internal standard. The samples then underwent acid hydrolysis in 2 mL 0.5 M HCl in acetonitrile for 45 min at 100 °C followed by alkaline hydrolysis with 2 mL of 1 mol/L NaOH in methanol for 45 min at 100 °C. After samples were cooled down at room temperature, they were acidified with 250 µL 6 M HCl followed by two-stage fatty acid hexane extraction with 2 mL hexane. The hexane solution was dried down using a stream of nitrogen. The fatty acids were then derivatised using 50 µL MTBSTFA + 1% TBDMS for 30 min at 60 °C. For quantitation, a calibration line was created using 2-hydroxyphytanic acid and phytanic acid with heptadecanoic-17,17,17-d3 acid as the internal standard.

### 2.5. GC-MS Fatty Acid Analysis

An Agilent GC-MS system (GC 6890A and MS 5973) was used equipped with an HP-1MS capillary column (30 m × 250 µm i.d., 0.25µm film thickness). The GC temperature program was as follows: Initially 120 °C increased to 200 °C at a rate of 20 °C/min followed by 2 °C/min increase rate to 300 °C with a total run time of 60 min. Samples were injected at a temperature of 250 °C in splitless mode. Data were acquired by scan mode (m/z range 50–700) and selected ion monitoring (SIM). The SIM masses were as follows: Pentadecanoic acid-TBDMS = m/z 299; Heptadecanoic acid-TBDMS = m/z 327; D3-heptadecanoic acid-TBDMS = m/z 330; Phytanic acid-TBDMS = m/z 369; 2-hydroxyphytanic acid-TBDMS = m/z 499.

### 2.6. Preparation of Mouse Liver, Kidney, and Brain Tissues for Proteomic Analysis

Fresh frozen mouse tissues (wild-type *n* = 3; *Hacl1^−^*^/*−*^ *n* = 3 per tissue) were homogenised in 500 µL homogenization buffer (100 mmol/L Tris, 6 mol/L urea, 2 mol/L thiourea, 2% ASB-14, pH 7.8) with protease inhibitor cocktail using a Minilys tissue homogeniser. Tissues were homogenised for 4 × 30 s intervals while placed on ice for 30 s in between intervals. Samples were then centrifuged for 30 s and the supernatant transferred to 1.5 mL Eppendorf tubes for further centrifugation at 14,000× *g* for 30 min at 4 °C. A hundred microliters of the supernatant were then used for chloroform/methanol protein precipitation with the remaining homogenate stored at −80 °C.

### 2.7. Protein Precipitation

With a starting volume of 100 µL homogenate, 400 µL methanol were added and mixed followed by addition of 100 µL chloroform and mixing, and finally topped up with 300 µL H_2_O. The solution was centrifuged at 14,000× *g* for 2 min after which the top aqueous layer was discarded. Further 400 µL methanol were added and mixed then centrifuged at 14,000× *g* for 3 min. The methanol was discarded and the samples were dried by SpeedVac. The pellets were resuspended in 100 µL digest buffer (100 mmol/L Tris, 6 mol/L urea, 2 mol/L thiourea, 2% ASB-14, pH 7.8) and shaken for 2 h at room temperature.

### 2.8. Protein Digestion

Twenty microliters of lysate were reduced with 1.5 uL of 195 mmol/L 1,4-Dithioerythritol for 30 min at 37 °C followed by alkylation with 195 mmol/L iodoacetamide for 30 min at room temperature in darkness. The solution was diluted with 155 µL deionised H_2_O and 20 µL of 1 µg/10 µL LysC/Trypsin were added. The samples were incubated for 24 h at 37 °C with gentle shaking. The peptides were desalted by C18 cartridge solid phase extraction and lyophilised before reconstitution in 200 µL 3% Acetonitrile + 0.1% Formic acid solution.

### 2.9. LC-MS Proteomic Analysis

Samples were analysed using a QToF SYNAPT G2-Si mass spectrometer coupled to a NanoAcquity UPLC system (Waters Corp, Wilmslow, UK) using the previously described method [14]. Briefly, the liver and kidney peptide samples were analysed using an on-line two dimensional high/low pH LC setup. An amount of 4 µL of peptide solution was injected and fractionated on an XBridge Peptide ethylene bridged hybrid C18 NanoEase Column (130 Å, 5 μm, 300 μm × 50 mm) using mobile phase A of 20 mmol/L ammonium formate, pH 9 and mobile phase B of acetonitrile. The sample was fractionated by injecting into the XBridge Peptide column with 3% mobile phase B, and then using a 4 min gradient up to the following mobile phase B percentages (liver, 4 fractions: 11.8%, 15.3%, 19.3% and 50%; kidney, 6 fractions: 10.1%, 13.1%, 15.3%, 17.7%, 21.2%, and 70%). Following elution from the XBridge peptide column, each fraction entered an ACQUITY UPLC Peptide ethylene bridged hybrid C18 nanoACQUITY Column (10 Kpsi, 130 Å, 1.7 μm, 75 μm × 150 mm) (Waters Corp, Wilmslow, UK) at a flow of 400 nL/min and maintained at 35 °C, and was then eluted with mobile phase A of 0.1% *v*/*v* formic acid with 5% *v*/*v* dimethyl sulphoxide and mobile phase B of 0.1 % *v*/*v* formic acid in 100% acetonitrile with 5% *v*/*v* dimethyl sulphoxide. The gradient started at 3% mobile phase B reaching 40% after 40 min, increasing to 85% by minute 42 and kept for 2 min before returning to 3% at 45 min until 60 min. The brain peptides were analysed by one dimensional LC setup by injection into the Peptide ethylene bridged hybrid C18 column without the fractionation step.

### 2.10. Proteomic MS Data Analysis 

The mass spectra were analysed using Waters Progenesis QI for Proteomics software. Protein identifications were accepted with 2 peptides including 1 unique peptide, or at least 3 peptides with no unique peptide. Protein identities were searched against a mouse proteins database downloaded from uniport.org. Peptide identification parameters allowed for 2 missed cleavages, a maximum protein mass of 800 kDa, and modifications of C carbamidomethylation, N and Q deamidation, M oxidation, and pyrrolidone carboxylate. The false detection rate was set to less than 4%, with ion matching requirements of 3 fragments per peptide, 5 fragments per protein, and 1 peptide per protein. Normalisation of ion abundance across the samples was performed using Progenesis QI. Differentially expressed proteins were chosen based on a minimum 1.5-fold change and *p*-value < 0.01. Protein–protein interaction, KEGG pathway analysis, and gene ontology enrichment analysis were performed using Metascape.org [15] and DAVID Bioinformatics database 6.8. The protein networks were mapped using Cytoscape (v3.8.2).

### 2.11. Statistical Analysis and Data Plotting

All statistical tests were performed using OriginPro 2020 v9.7 (OriginLab Corp, Northampton, MA, USA). Graphs were plotted using OriginPro 2020 v9.7, Microsoft Excel (2016), and VolcaNoseR [16].

## 3. Results

### 3.1. Hacl1^−/−^ Mouse Phenotype

*Hacl1*^−/−^ mice did not show Hacl1 protein in liver, kidney, brain, or heart when analysed by Western blot (Figure S1). The *Hacl1*^−/−^ mice were apparently healthy, with no obvious phenotypic abnormalities and with no differences from the wild-type littermate controls observed, with or without a phytol-enriched diet. Over a 6-week period, no consistent differences in body weight, markers of renal function (Figure S2), acoustic brainstem (Figure S3A), fear conditioning (Figure S3B) or locomotor activity responses (Figure S3C) were observed between the wild-type and knockout animals. Optical coherence tomography revealed a similar proportion of homozygotes (12/26) compared with controls (4/14) with abnormalities (*p* = 0.33) (Figure S4).

### 3.2. Fatty Acid Profile in Hacl1^−/−^ Mouse Liver

**Figure 2:**
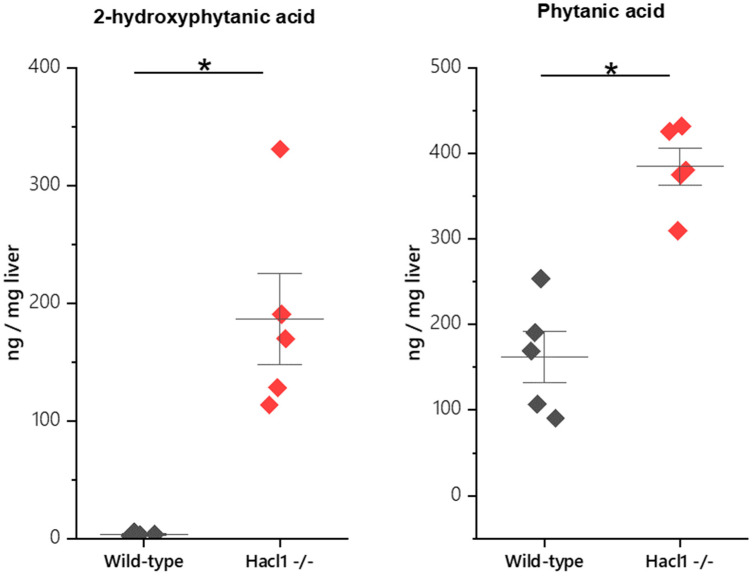
Phytanic acid and 2-hydroxyphytanic acid concentrations in mouse liver. Both fatty acids are significantly elevated in *Hacl1*^−/−^ liver (* indicates *p* < 0.01).

Phytanic acid and 2-hydroxyphytanic acid concentrations were measured in the livers of wild-type and *Hacl1*^−/−^ mice fed a high phytol diet. Both fatty acids were significantly elevated in *Hacl1*^−/−^ liver (*p* < 0.01) (Figure 2). In contrast, the odd chain fatty acid heptadecanoic acid was significantly reduced (*p* < 0.01) (Figure S5). These are in agreement with previously reported findings in Hacl1 deficient mouse liver [11].

### 3.3. Hacl1^−/−^ Mouse Liver Proteome

**Figure 3:**
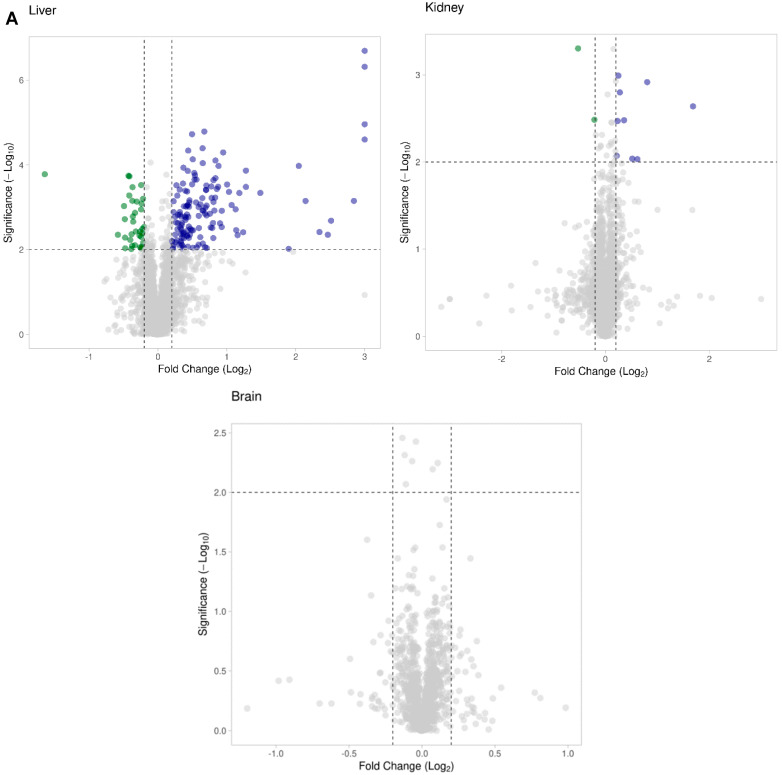

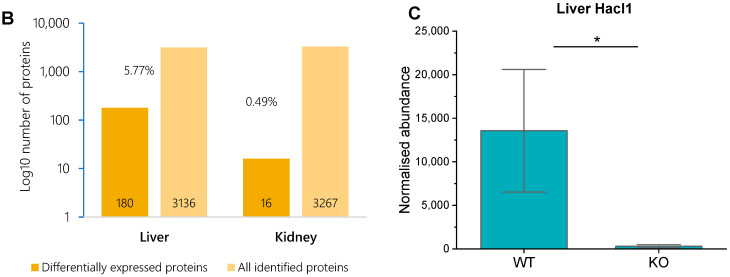
Proteomic analysis of *Hacl1^−^*^/*−*^ mouse tissue. (**A**) Scatter plot of proteins identified and their fold change in *Hacl1*^−/−^ liver, kidney, and brain tissue compared to wild-type. Proteins with 1.5-fold change (*p* < 0.01) were chosen as significantly differentially regulated. (**B**) Percentage of differentially expressed proteins in *Hacl1*^−/−^ liver and kidney. (**C**) Hacl1 peptide abundance in mouse liver (* indicates *p* < 0.01).

We compared the liver proteome of wild-type and *Hacl1*^−/−^ mice fed a high phytol diet. A total of 3136 proteins were identified, of these, 2646 proteins with at least 1 unique peptide. Using Progenesis QI for proteomics, the normalised abundance of each protein was compared in the wild-type and *Hacl1*^−/−^ and 180 proteins were found to be differentially regulated by a minimum of 1.5-fold (*p* < 0.01) (Figure 3A,B). Of these, 142 proteins were upregulated, and 38 proteins were downregulated in *Hacl1*^−/−^ liver (Figure S6). The Hacl1 peptides were identified in both wild-type and knockout livers and with a 44-fold decrease in normalised abundance in the knockouts (Figure 3C). As the Hacl1 protein was not detected by Western blot in the knockouts (Figure S1), the presence of these peptides is likely as a result of leaky expression from the knockout cassette or the presence of a low amount of full-length or truncated protein produced by exon skipping.

**Figure 4:**
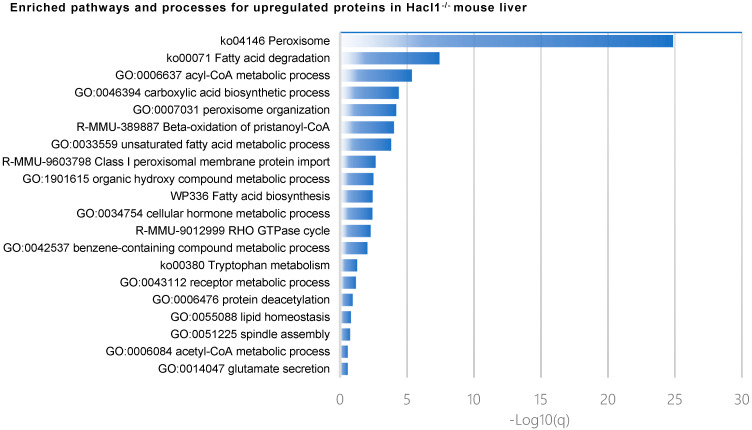

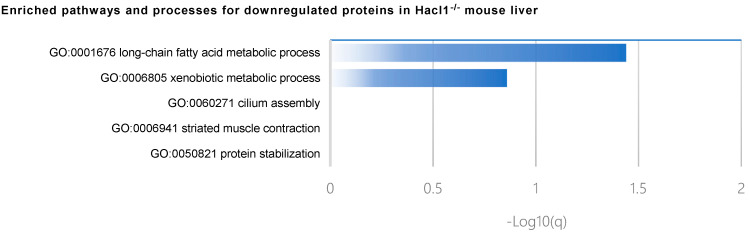
Gene ontology and pathway analysis of upregulated and downregulated proteins in *Hacl1*^−/−^ mouse liver.

Metascape analysis of pathway and process enrichment revealed that biological processes such as the Acyl-CoA metabolic process, carboxylic acid biosynthetic process, peroxisome organisation, and unsaturated fatty acid metabolic process were enriched in upregulated proteins (Figure 4). The KEGG pathway analysis of the upregulated proteins revealed 25 proteins were associated with metabolic pathways; of these, 22 and 8 proteins were associated with the peroxisome and PPAR signalling pathway, respectively, with statistical significance (*p* < 0.01) (Table 1). Biological processes enriched in the downregulated proteins include the long-chain fatty acid metabolic process, xenobiotic metabolic process, and cilium assembly (Figure 4).

**Figure 5:**
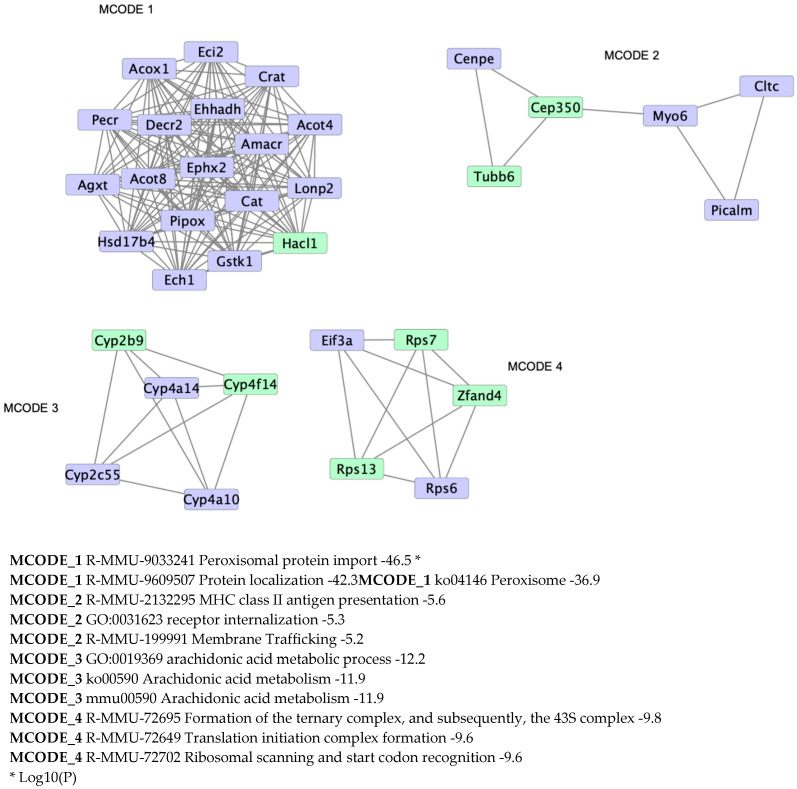
Protein–protein interaction enrichment analysis showing physical interactions formed among the upregulated (blue) and downregulated (yellow) proteins in *Hacl1*^−/−^ liver.

A protein–protein interaction enrichment analysis was performed on differentially regulated proteins. The upregulated proteins mainly form one large cluster associated with peroxisomal protein localisation as part of the peroxisome pathway (Figure 5). Three other clusters are formed associated with the arachidonic acid metabolism, membrane trafficking, and translation (Figure 5).

Three Cyp4a proteins involved in ω-oxidation were identified, as follows: Cyp4a10, Cyp4a12a/b, and Cyp4a14. Only Cyp4a10 and Cyp4a14 were upregulated in the knockout liver, whereas Cyp4a12 expression was not significantly different. The Cyp4a proteins are also associated with the arachidonic acid and retinol metabolism pathways which were both enriched and also share Cyp2c55 (Table 1). In the arachidonic acid metabolism pathway, Ephx2, and Plb1 were also upregulated while Cyp2b9 and Cyp4f14 were downregulated (Table 1).

### 3.4. Hacl1^−/−^ Mouse Kidney Proteome

A total of 3267 proteins were identified in the kidneys with 16 proteins significantly differentially regulated by at least 1.5-fold (*p* < 0.01) (Figure 3A,B). Of these, 13 proteins were upregulated and 3 were downregulated in the *Hacl1*^−/−^ kidney. Gene ontology enrichment analysis revealed five of the upregulated proteins Scp2, Zadh2, Hsd17b4, Crot, and Acsl3 are peroxisomal (Table 2). The KEGG pathway analysis revealed enrichment of the peroxisome, primary bile acid biosynthesis and PPAR signaling pathways (Table 2). The protein–protein interaction enrichment analysis of the upregulated proteins showed the formation of a network between Acsl3, Hsd17b4, and Scp2 associated with Beta-oxidation of pristanoyl-CoA (R-MMU-389887), peroxisomal lipid metabolism (R-MMU-390918), and peroxisomal protein import (R-MMU-9033241) (Figure S7). Hacl1 peptides were identified in the wild-type but with lower abundance than in liver. However, an identification was also made of these peptides in some of the knockout kidneys with no significant difference in abundance with the wild-type. The Hacl1 protein was not detected by Western blot in the knockouts (Figure S1). Hence, we suspect that the detection of these peptides in the knockouts is explained similarly as those detected in the knockout liver. Cyp4a10 and Cyp4a14 were also detected in both groups but with no significant difference in their expression.

### 3.5. Hacl1^−/−^ Mouse Brain Proteome

A total of 1434 proteins were identified in the mouse brains and there were no significant differences in their expression in the wild-type and *Hacl1*^−/−^ groups (Figure 3A). Neither Hacl1 nor the ω-oxidation Cyp4a proteins were identified in either group. 

## 4. Discussion

In agreement with the results obtained by Mezzar et al. [11], this study found that Hacl1 deficient mice fed a high phytol diet had significantly higher hepatic levels of phytanic and 2-hydroxyphytanic acid and significantly lower hepatic levels of heptadecanoic acid (C17:0) than controls fed the same diet. The mean phytanic acid content (expressed as ng/mg liver) was 2.4-fold higher, whereas the mean 2-hydroxyphytanic acid was 55-fold higher. This is consistent with defective α-oxidation of phytanate at the level of 2-hydroxyacyl-CoA ligase. In contrast, in Refsum disease, there is no accumulation of 2-hydroxyphytanic acid [17]. It therefore behoves us to consider whether the profound changes in gene expression revealed by the liver proteomic analysis owes more to the accumulation of 2-hydroxyphytanic acid than that of phytanic acid. Studies of 2-hydroxyphytanic acid as a PPARα agonist would be interesting. Furthermore, the low content of heptadecanoic acid in the *Hacl1***^−/−^** livers is consistent with significant synthesis of odd-chain fatty acids from 2-hydroxyfatty acids [11].

*Hacl1* knockout mice did not develop any notable phenotypes in our investigation compared with Mezzar et al. [11], in which they observed weight loss, an enlarged mottled liver, reduced hepatic glycogen and triglycerides, and no abdominal white adipose tissue. These outcomes could be explained by the different background strain used in our study that would account for most of the variation. In addition, mice in our study were fed a lower phytol diet (0.2% *w*/*w*) over 10 weeks in their home cage, which may also attribute to the absence of phenotypes found herein.

In this study, we have used a proteomics approach to study the activation of an alternative pathway for phytanic acid oxidation through upregulation of cytochrome P450 enzymes involved in fatty acid ω-oxidation, peroxisome proliferation and upregulation of a transporter and enzymes involved in β-oxidation of dicarboxylic acids. This upregulation also correlates with the reported identification of metabolic intermediates of phytanic acid ω- and β-oxidation in *Hacl1***^−/−^** mice [11]. Phytanic acid is known to activate PPARα [18], thus it is reasonable to expect its accumulation will induce ω-oxidation, peroxisome proliferation and increased expression of peroxisomal β-oxidation enzymes. In Refsum patients, ω- plus β-oxidation of phytanic acid is evident by the detection of urinary 3-methyladipic acid [19]. It remains unclear if accumulating 2-hydroxyphytanic acid can also act as a PPARα agonist or as an agonist for RXR, the PPAR co-activator.

The first step in the alternative pathway is ω-oxidation. This study showed that in the livers of phytol-fed *Hacl1***^−/−^** mice there was upregulation of the ω-oxidation enzymes Cyp4a10 and Cyp4a14 but not Cyp4a12. This is consistent with the upregulation being mediated by PPAR signaling. It has been shown in mouse liver and kidney that *Cyp4a12* gene expression, unlike *Cyp4a10 and Cyp4a14*, is not induced by fibrates [20]. After generation of dicarboxylic acids by the Cyp4a10 and Cyp4a14, the next step in the alternative pathway is the formation of the dicarboxylyl-CoA ester, which then needs to be transported into the peroxisomes. The transporter that accomplishes import of dicarboxylyl-CoA esters and branched chain acyl-CoA esters is Abcd3 [21,22]. In this study, Abcd3 was upregulated in the livers of *Hacl1***^−/−^** mice fed a high phytol diet. Peroxisomal β-oxidation of dicarboxylic acids is catalysed by acyl-CoA oxidase 1 (Acox1), the peroxisomal L-bifunctional protein and the Acaa1b thiolase [22]. Expression of all three enzymes was upregulated in the livers of *Hacl1***^−/−^** mice fed a high phytol diet. The export of medium chain dicarboxylyl-CoA esters from the peroxisomes is achieved by conversion to carnitine by the peroxisomal carnitine O-octanoyl transferase (Crot). This study showed that expression of this enzyme was increased in the livers of *Hacl1***^−/−^** fed a high phytol diet.

In summary, therefore, a proteomic analysis of livers from *Hacl1***^−/−^** and *Hacl1^+^*^/*+*^ mice fed a high phytol diet has shown that every protein required for the oxidation of phytanic acid by the alternative pathway of ω-oxidation followed by peroxisomal import, peroxisomal β-oxidation and peroxisomal export is upregulated in the *Hacl1***^−/−^** animals. There is strong evidence for the involvement of PPARα in this process. Phytanic acid is a known PPARα agonist and the level of phytanic acid was 2.4-fold higher in the *Hacl1***^−/−^** livers. There was a much more substantial build-up of 2-hydroxyphytanic acid in the *Hacl1***^−/−^** livers (55-fold) but whether this compound activates PPARα or an X receptor (such as RXR) is not known.

It was not only the alternative pathway for phytanic acid oxidation that was differentially regulated in *Hacl1***^−/−^** livers. Several proteins associated with the arachidonic acid (AA) metabolism pathway were also differentially regulated. It was recently shown that the PPARα agonist, clofibrate, increases molecular species of phosphatidylethanolamine containing arachidonic acid for biogenesis of peroxisomal membranes in peroxisome proliferation in the liver [23]. The downregulated monooxygenase Cyp4f14, orthologue for human CYP4F12, is involved in the ω-hydroxylation of arachidonic acid to 20-hydroxyeicosatetraenoic acid [24], while Cyp2b9, also downregulated, is the orthologue for humans CYP2B6, and involved in arachidonic acid epoxidation [25]. Thus, downregulation of both these enzymes could reduce catabolism of arachidonic acid allowing for increased synthesis of phosphatidylethanolamines containing AA. For Cyp4f14, the mechanism may not involve a direct effect of PPARα since, although PPARα regulates CYP4A enzymes, it does not have control over the CYP4F enzymes. Instead, CYP4F12 is regulated by pregnane-X receptor (PXR) in human hepatocytes [26]. On the other hand, CYP2B6 can be regulated by PPARα, PXR, and constitutive androstane receptor (CAR) [25,26,27]. Many exogeneous and endogenous ligands have been identified as activators and few as inhibitors of PXR and CAR [28]. These include stigmasterol and docosahexaenoic acid which inhibit PXR and CAR activity, respectively. However, Cyp2c55 is also regulated by PXR and CAR and is upregulated in *Hacl1***^−/−^** liver. Thus, it seems unlikely that PXR and CAR activity is partially inhibited in *Hacl1***^−/−^**mice on a high phytol diet and suggests Cyp4f14 and Cyp2b9 suppression is not PXR and/or CAR dependent. Nevertheless, further investigation on the effect of diterpenoids and phytanic acid on PXR and CAR activity is needed. The *Hacl1****^−^*^/*−*^** mice showed upregulation of two enzymes involved in AA metabolism. Upregulation of phospholipase B1 (Plb1) would be consistent with increased supply of AA from phosphatidylcholines to allow for synthesis of phosphoethanolamines containing AA. The reason for upregulation of the epoxide hydrolase (Ephx) is not clear.

The kidney proteome indicated an insignificant difference in the expression of Hacl1 in the wild-type and *Hacl1***^−/−^** mouse, expectedly since the liver peroxisomes are the main site for α-oxidation and Hacl1 expression is relatively low in kidney peroxisomes [29]. Moreover, in contrast to the liver, *Hacl1***^−/−^** kidney expression of Cyp4a10 and Cyp4a14 was not elevated suggesting ω-oxidation is not necessarily activated. One possible explanation is the presence of the peroxisomal hydroxyacid oxidase 2 (Hao2) which is mainly expressed in kidneys and is active towards 2-hydroxypalmitic acid converting it to 2-oxo-palmitic acid [30], with possible activity towards 2-hydroxyphytanic acid [31]. However, there was no significant difference in Hao2 expression in *Hacl1***^−/−^** kidney. This and the low expression of Hacl1 in kidney peroxisomes suggests Hao2, and not Hacl1, is primarily active in 2-hydroxyphytanic acid oxidation. The Hao2 activity has been shown to be comparable in male and female *Hacl1***^−/−^** kidney on high phytol diet [11]. On the other hand, an endoplasmic reticulum lyase resembling bacterial acetolactate synthase has been recently described. It shares substrate specificity with HACL1 and has been reported to have higher activity in *Hacl1***^−/−^** kidney compared to liver [11]. However, peptides for this lyase were not identified in our kidney analysis of both wild-type and knockouts. Since only 13 proteins were significantly upregulated in the *Hacl1***^−/−^** kidney, a few GO processes and KEGG pathway terms were enriched. The upregulated peroxisomal proteins Acsl3, Crot, Hsd17b4, and Scp2 are mediated by PPARα [32,33]. 

The *Hacl1***^−/−^** brain proteome showed no significant difference in protein expression. More so, Hacl1 was not detected, which is in agreement with published data that shows Hacl1 mRNA to be virtually undetectable in the mouse brain [34]. None of the Cyp4a proteins detected and elevated in the liver where detected in the brain, which is also consistent with mRNA expression studies showing mouse brain Cyp4a10, Cyp4a12, and Cyp4a14 relative expression to be very low [35]. Cerebronic acid (2OH-C24:0) is a major 2-hydroxylated fatty acid component of cerebrosides and sulfatides in the brain and nervous tissues that undergoes peroxisomal α-oxidation. However, the enzymes involved in cerebronic acid α-oxidation are distinct from those involved in phytanic acid α-oxidation. As a result, Refsum disease patient fibroblasts do not show an impairment of cerebronic acid α-oxidation [36]. Similarly, *Hacl1***^−/−^** mice fed a phytol diet show no change in their fatty acid profile [11]. Both the levels of the major 2-hydroxylated fatty acids exclusively located in nervous tissues and the odd chain fatty acids are unaffected in these mice. This, along with our proteomic findings, further support the existence of an alternate α-oxidation pathway for 2-hydroxylated fatty acids in the brain with an acyl-CoA lyase distinct from HACL1 [11,36].

Mutations directly associated with the peroxisomal fatty acid α-oxidation pathway are extremely rare, with an estimated 1 in 10^6^ incidence of Refsum disease in the UK [37], while only a handful of AMACR deficient patients have been reported in the literature. So far, there has been no definite report of a HACL1 deficient patient, although HACL1 was one of 57 candidate genes for a recessive syndrome involving intellectual disability, muscle weakness and a characteristic face [38]. However, we speculate that the alternative pathway activated in Hacl1 deficient mice may also be activated in humans, resulting in the lack of a clear peroxisomal metabolic disorder phenotype, hence the absence of an identified case.

In summary, this study provides an insight into the mechanisms by which an alternative pathway is activated as a result of Hacl1 deficiency and validates previous findings on PPARα and Cyp4a involvement. Discovery proteomic studies are lacking in Refsum and Amacr-deficient mouse models thus studying their proteome could provide better understanding of disease mechanism and potentially drug targeting. This study is intended as a resource for further investigation into disorders associated with peroxisomal fatty acid alpha-oxidation and demonstrates the power of metabolomic and proteomic approaches in studying metabolic pathways.

## Figures and Tables

**Table 1:** KEGG pathway analysis of upregulated proteins in *Hacl1*^−/−^ mouse liver.

KEGG Pathway	Count	*p*-Value	Gene
Peroxisome	22	1.60 × 10^−25^	Pecr, Gstk1, Acot8, Pex19, Abcd3, Acsl1, Pex11a, Ephx2, Eci2, Ech1, Hsd17b4, Pipox, Acaa1b, Acaa1a, Amacr, Acox1, Ehhadh, Cat, Decr2, Agxt, Crat, Nudt12
Fatty acid degradation	9	1.30 × 10^−8^	Aldh3a2, Acsl1, Acox1, Ehhadh, Cyp4a10, Eci2, Acaa1b, Cyp4a14, Acaa1a
Biosynthesis of unsaturated fatty acids	6	4.30 × 10^−6^	Pecr, Acox1, Acaa1b, Scd1, Acaa1a, Acot4
Fatty acid metabolism	7	7.00 × 10^−6^	Pecr, Acsl1, Acox1, Ehhadh, Acaa1b, Scd1, Acaa1a
PPAR signaling pathway	8	8.60 × 10^−6^	Acsl1, Acox1, Ehhadh, Cyp4a10, Acaa1b, Cyp4a14, Scd1, Acaa1a
Metabolic pathways	25	3.40 × 10^−4^	Hsd17b4, H2-Ke6, Acaa1b, Acaa1a, Plb1, Cyp2c55, Rdh16, Dlat, Acot8, Acsl1, Ephx2, Cyp4a10, Pmm2, Pipox, Cyp4a14, Kmo, Aldh3a2, Amacr, Acox1, Ehhadh, Agxt, Gart, Mgll, Acot4, Uox
alpha-Linolenic acid metabolism	4	1.60 × 10^−3^	Acox1, Acaa1b, Acaa1a, Plb1
Valine, leucine and isoleucine degradation	5	1.70 × 10^−3^	Aldh3a2, Ehhadh, Acaa1b, Acaa1a, Aacs
Arachidonic acid metabolism	5	9.50 × 10^−3^	Cyp2c55, Ephx2, Cyp4a10, Cyp4a14, Plb1
Tryptophan metabolism	4	9.50 × 10^−3^	Aldh3a2, Ehhadh, Cat, Kmo
Primary bile acid biosynthesis	3	9.50 × 10^−3^	Acot8, Amacr, hsd17b4
Lysine degradation	4	1.30 × 10^−2^	Aldh3a2, Setd2, Ehhadh, Pipox
Retinol metabolism	4	5.00 × 10^−2^	Cyp2c55, Cyp4a10, Rdh16, Cyp4a14
Pyruvate metabolism	3	5.10 × 10^−2^	Aldh3a2, Acot12, Dlat
Carbon metabolism	4	9.50 × 10^−2^	Ehhadh, Cat, Dlat, Agxt

**Table 2:** Gene Ontology and KEGG pathway analysis of upregulated proteins in *Hacl1***^−/−^** mouse kidney.

	*p*-Value	Genes
**GO Biological Process**		
Fatty acid metabolic process	2.60 × 10^−3^	Acsl3, Crot, Hsd17b4
Lipid metabolic process	2.10 × 10^−2^	Acsl3, Crot, Hsd17b4
Fatty acid beta-oxidation	2.20 × 10^−2^	Crot, Hsd17b4
**GO Cellular Compartment**		
Peroxisome	6.00 × 10^−7^	Acsl3, Crot, Hsd17b4, Scp2, Zadh2
Intracellular membrane-bounded organelle	7.30 × 10^−3^	Acsl3, Crot, Hsd17b4, Scp2
Mitochondrion	1.20 × 10^−2^	Acsl3, Crot, Hsd17b4, Scp2, Zadh2
Peroxisomal membrane	2.90 × 10^−2^	Acsl3, Hsd17b4
**GO Molecular Function**		
Receptor binding	1.40 × 10^−2^	Crot, Hsd17b4, Scp2
Actin filament binding	5.90 × 10^−2^	Myo1b, Svil
Transferase activity, transferring acyl groups	7.40 × 10^−2^	Crot, Scp2
**KEGG Pathway**		
Peroxisome	1.20 × 10^−6^	Acsl3, Crot, Hsd17b4, Scp2
Primary bile acid biosynthesis	6.20 × 10^−3^	Hsd17b4, Scp2
PPAR signaling pathway	3.10 × 10^−2^	Acsl3, Scp2
Metabolic pathways	7.30 × 10^−2^	Acsl3, Hsd17b4, Scp2

## Data Availability

The raw mass spectrometric data has been deposited in the UCL Research Data Repository. The proteomics dataset can be accessed by visiting https://doi.org/10.5522/04/17025359, accessed on 15 December 2021.

## Supplementary Materials

The supporting information can be downloaded at: https://www.mdpi.com/article/10.3390/ijms23020987/s1.
