# Supplementary material for: Tissue Proteome of 2-Hydroxyacyl-CoA Lyase Deficient Mice Reveals Peroxisome Proliferation and Activation of ω-Oxidation
Source: Int J Mol Sci. 2022 Jan 17;23(2):987. doi: 10.3390/ijms23020987 (PMC8781152; doi:10.3390/ijms23020987)
Supplement: Supplementary file 1 [file ijms-23-00987-s001.zip › ijms-1535374-supplementary.pdf]

SUPPLEMENTARY INFORMATION

Tissue Proteome of 2-hydroxyacyl-CoA lyase deficient mice reveals peroxisome proliferation and activation of ω-oxidation

Youssef Khalil[1], Sara Carrino[1, 2], Fujun Lin[3, 4], Anna Ferlin[3, 5], Heena V Lad[6], Francesca Mazzacuva[1, 7], Sara Falcone[6], Natalie Rivers[6], Gareth Banks[6], Danilo Concas[6], Carlos Aguilar[6], Andrew R Haynes[6], Andy Blease[6], Thomas Nicol[6], Raya Al-Shawi[8], Wendy Heywood[1], Paul Potter[6], Kevin Mills[1], Daniel P Gale[3†], Peter T Clayton[1†*]

[1]Genetics and Genomic Medicine, Great Ormond Street Institute of Child Health, University College London, London WC1N 1EH, UK

[2]Department of Experimental, Diagnostic and Specialty Medicine, University of Bologna, Bologna 40138, Italy

[3]Department of Renal Medicine, University College London, London, NW3 2PF, UK

[4]Department of Nephrology, Xin Hua Hospital, School of Medicine, Shanghai Jiao Tong University, Shanghai, China

[5]Clinical Genetics and Genomics Laboratory, Royal Brompton Hospital, London SW3 6NP, UK

[6]MRC Harwell Institute, Harwell Campus, Oxfordshire, OX11 0RD, UK

[7]Department of Bioscience, University of East London, London E15 4LZ, UK

[8]Genetics Unit and Wolfson Drug Discovery Unit, Centre for Amyloidosis and Acute Phase Proteins, University College London, London, UK.

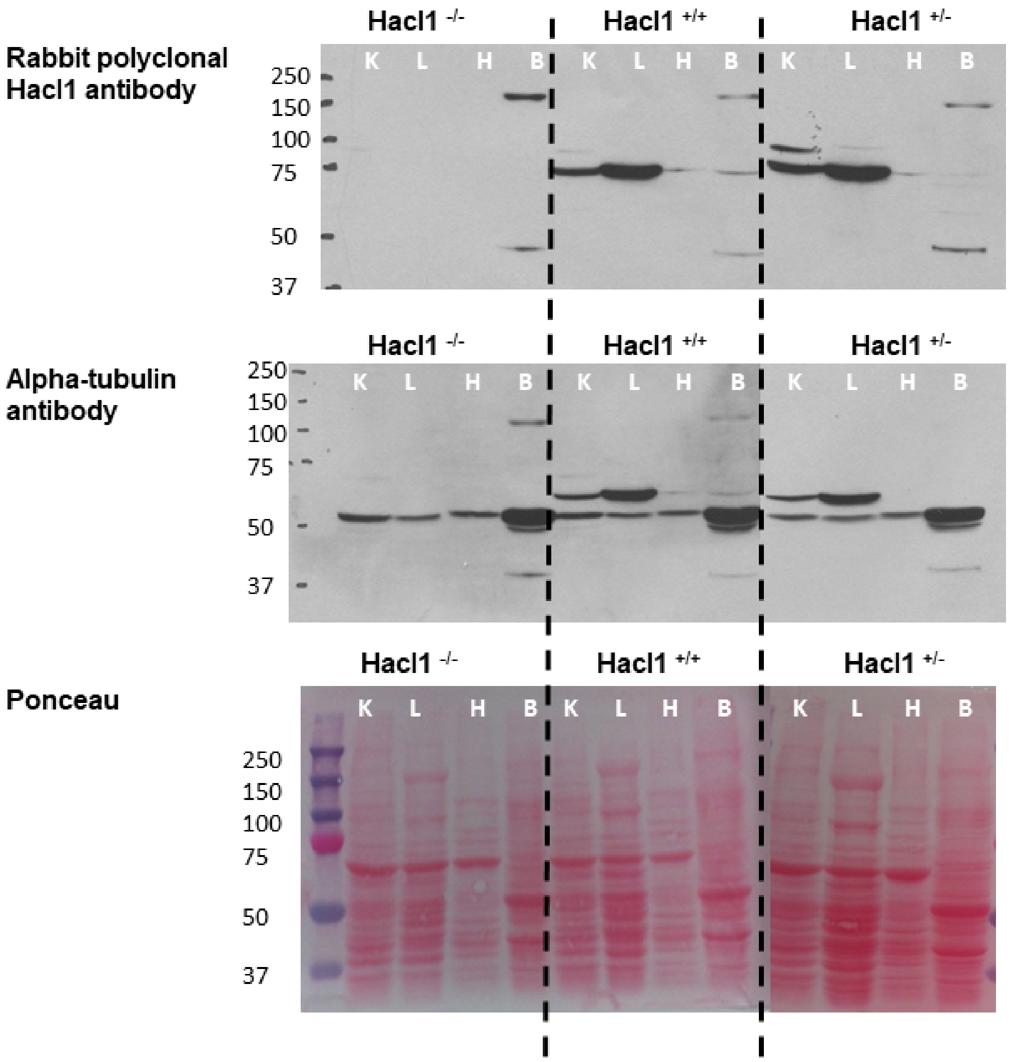

Figure S1. Western blots of mouse tissues. Hacl1 protein was not detected in knockout mouse tissue homogenates. K, Kidney; L, Liver; H, Heart; B, Brain

A

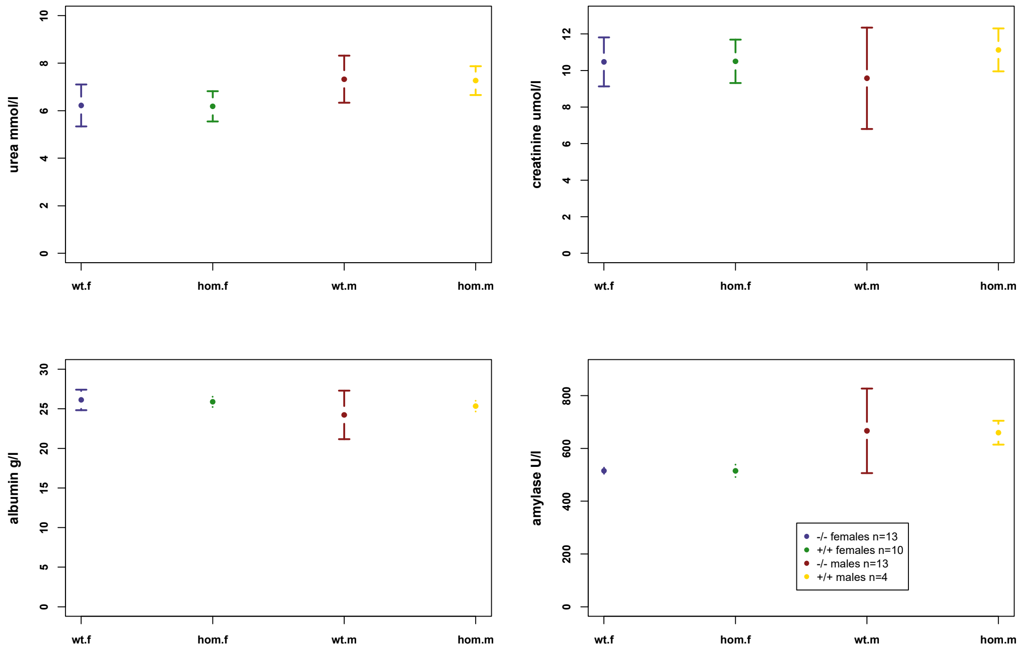

B

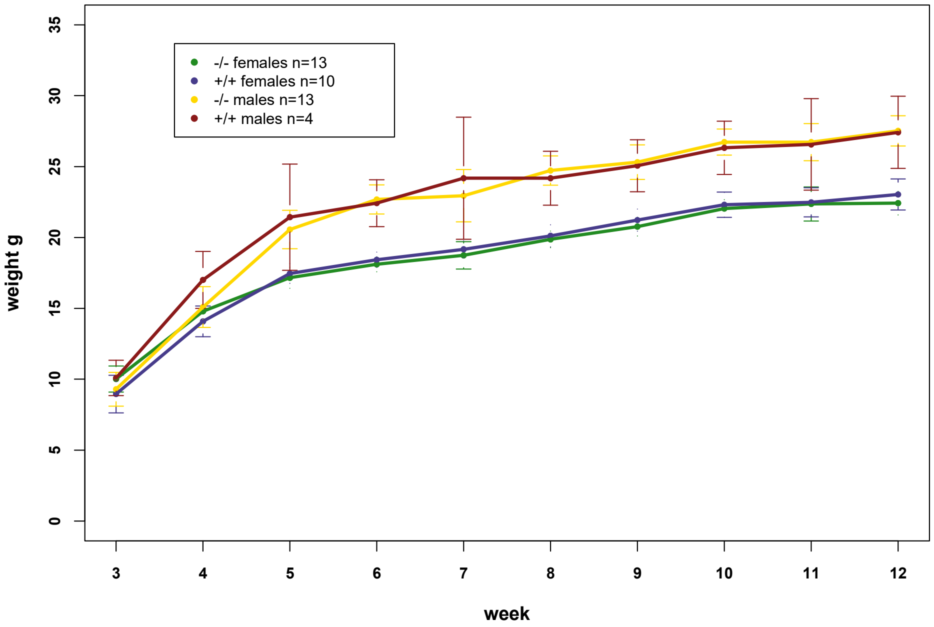

Figure S2. No differences in A) serum urea, creatinine, amylase or urine protein or B) body weight were observed between wild type and Hacl1-/- animals.

A

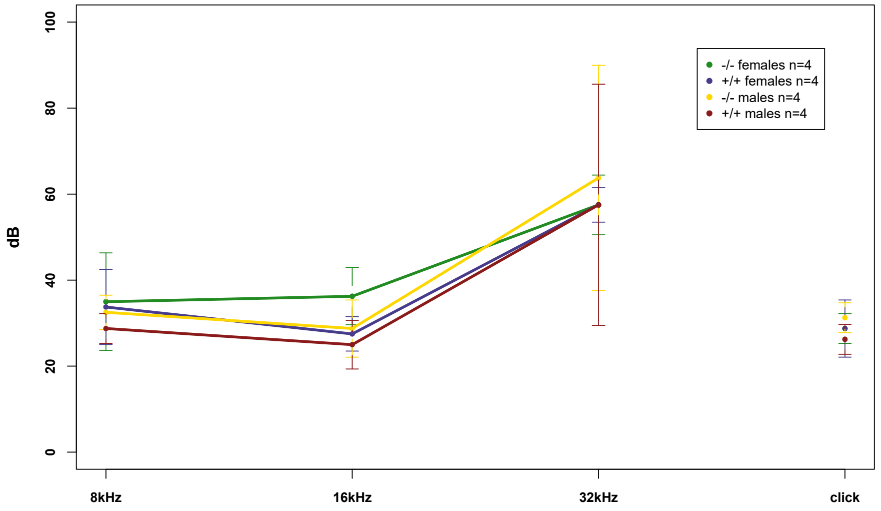

B

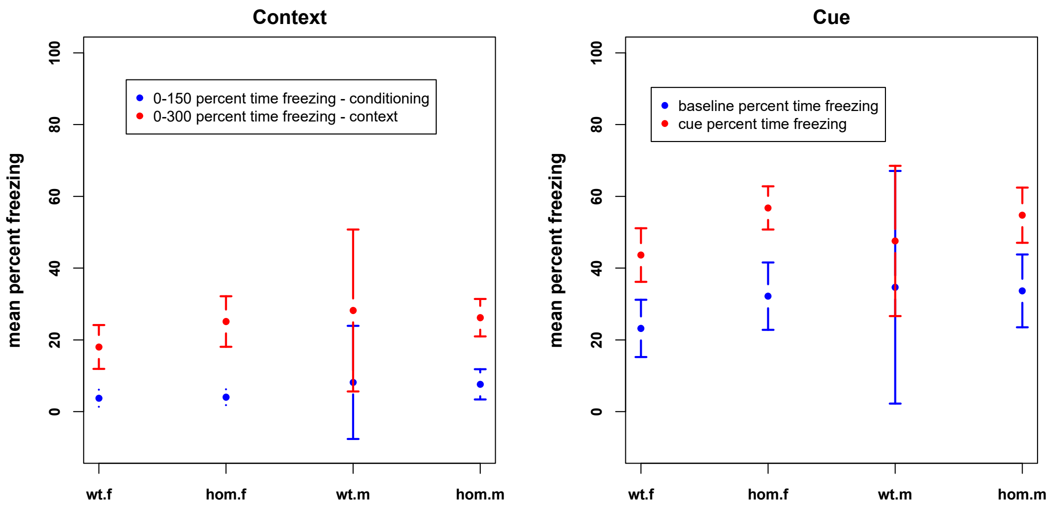

C

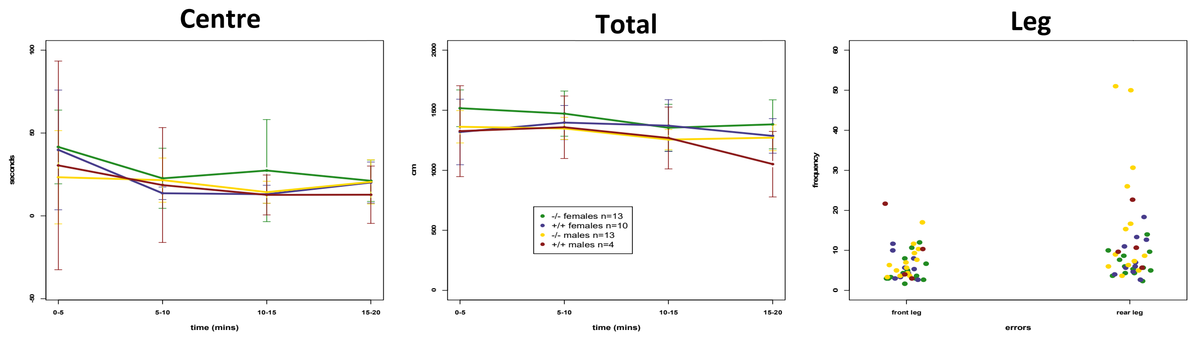

Figure S3. No differences in A) acoustic brainstem responses, B) fear conditioning responses or C) locomotor activity were observed between wild type and Hacl1-/- animals.

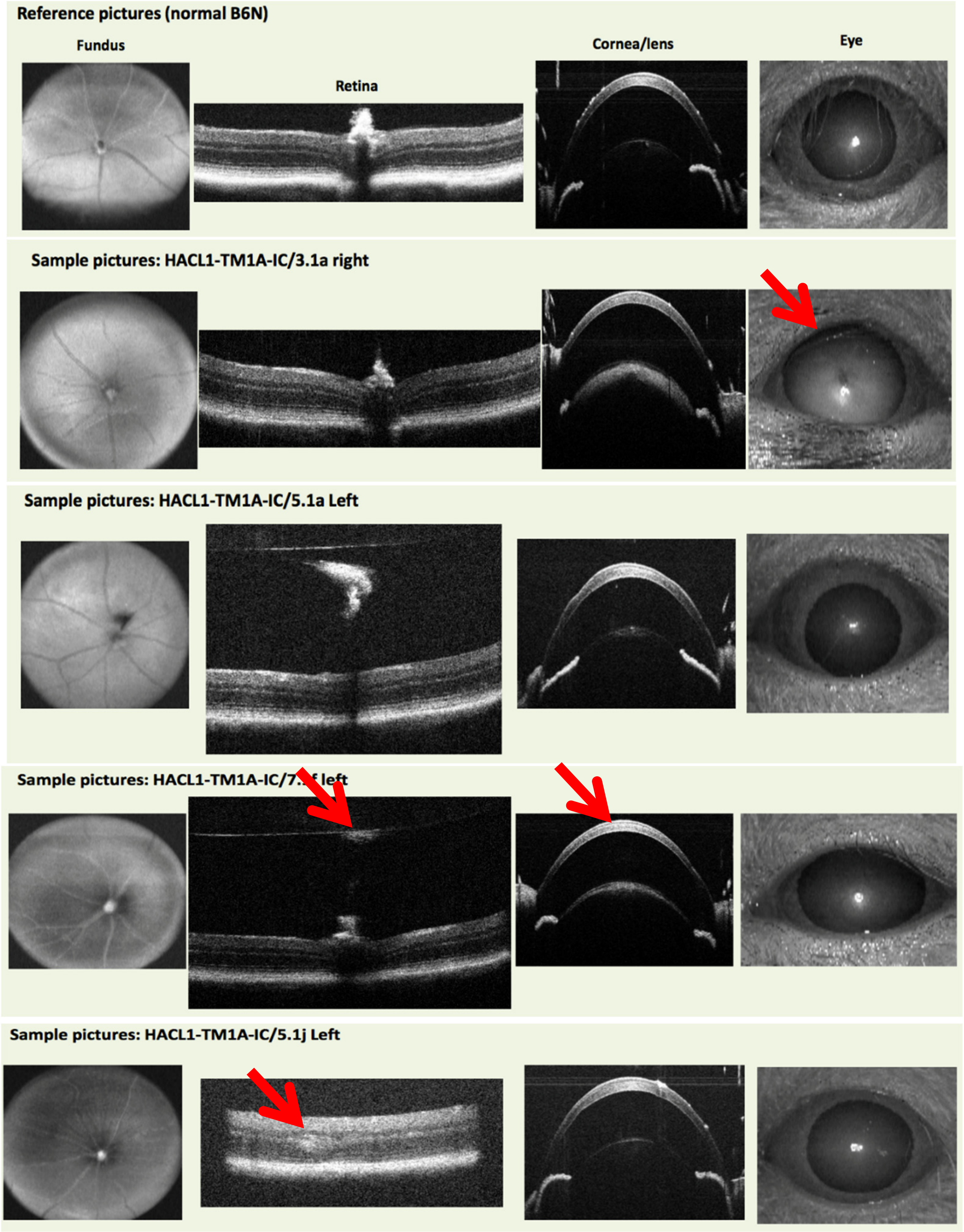

Figure S4. Representative examples of optical coherence tomography showing abnormalities (arrows) that were observed in both knockout than wild type animals.

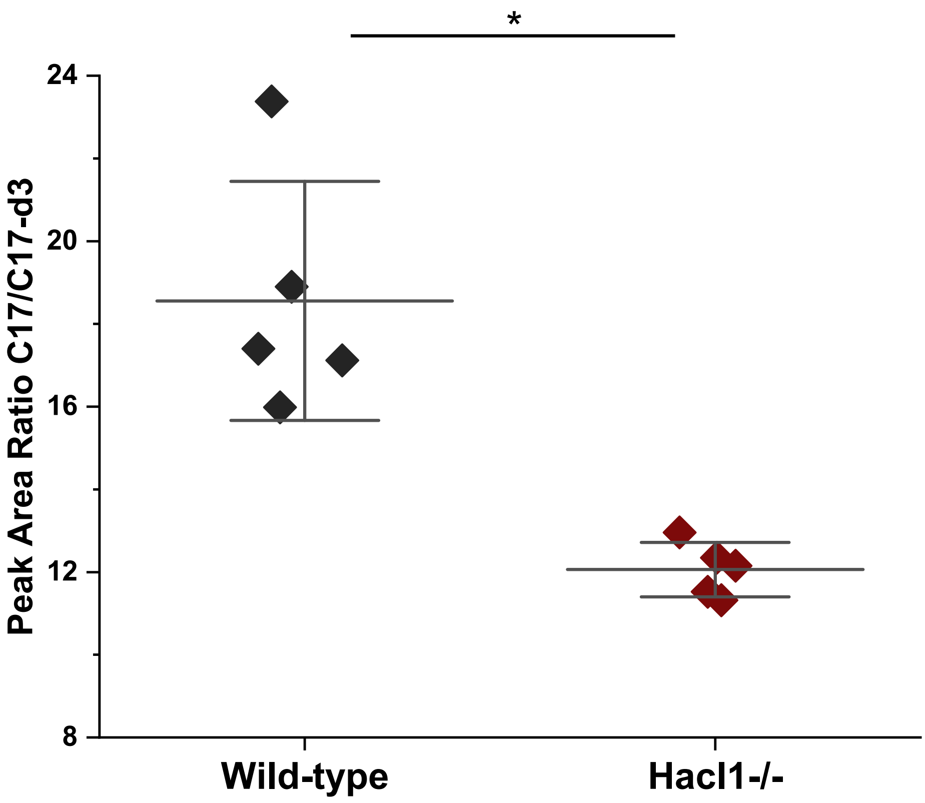

Figure S5. Liver heptadecanoic acid presented as peak area ratio with d3-heptadecanoic acid internal standard. Heptadecanoic acid is significantly reduced in *Hacl1*$^{-/-}$ liver (P<0.01).

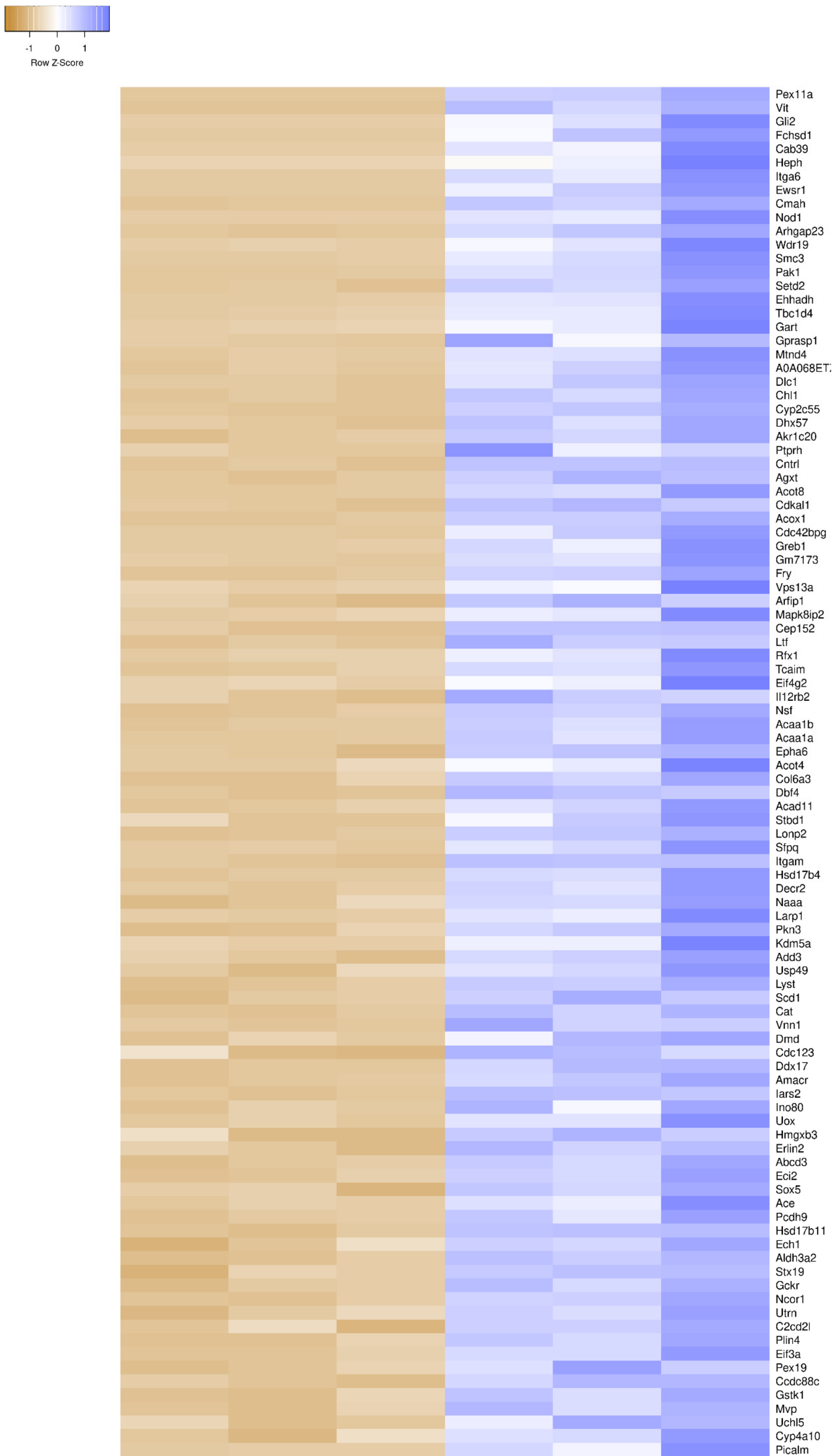
-1
0
1
Row Z-Score
Pex11a
Vit
Gli2
Fchsd1
Cab39
Heph
Itga6
Ewsr1
Cmah
Nod1
Arhgap23
Wdr19
Smc3
Pak1
Setd2
Ehhadh
Tbc1d4
Gart
Gprasp1
Mtnd4
A0A068ET
Dlc1
Chl1
Cyp2c55
Dhx57
Akr1c20
Ptprh
Cntrl
Agxt
Acot8
Cdkal1
Acox1
Cdc42bpg
Greb1
Gm7173
Fry
Vps13a
Arfip1
Mapk8ip2
Cep152
Ltf
Rfx1
Tcaim
Eif4g2
Il12rb2
Nsf
Acaa1b
Acaa1a
Epha6
Acot4
Col6a3
Dbf4
Acad11
Stbd1
Lonp2
Sfpq
Itgam
Hsd17b4
Decr2
Naaa
Larp1
Pkn3
Kdm5a
Add3
Usp49
Lyst
Scd1
Cat
Vnn1
Dmd
Cdc123
Ddx17
Amacr
Iars2
Ino80
Uox
Hmgxb3
Erlin2
Abcd3
Eci2
Sox5
Ace
Pcdh9
Hsd17b11
Ech1
Aldh3a2
Stx19
Gckr
Ncor1
Utrn
C2cd2l
Plin4
Eif3a
Pex19
Ccdc88c
Gstk1
Mvp
Uchl5
Cyp4a10
Picalm

MCODE 1

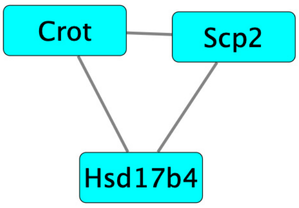

MCODE_1 R-MMU-389887 Beta-oxidation of pristanoyl-CoA
MCODE_1 R-MMU-390918 Peroxisomal lipid metabolism
MCODE_1 R-MMU-9033241 Peroxisomal protein import

Figure S7. Protein-protein interaction network in *Hacl1*$^{-/-}$ mouse kidney
